# Infrared LED Enhanced Spectroscopic CdZnTe Detector Working under High Fluxes of X-rays

**DOI:** 10.3390/s16101591

**Published:** 2016-09-27

**Authors:** Jakub Pekárek, Václav Dědič, Jan Franc, Eduard Belas, Martin Rejhon, Pavel Moravec, Jan Touš, Josef Voltr

**Affiliations:** 1 Institute of Physics, Faculty of Mathematics and Physics, Charles University, Ke Karlovu 5, Prague 12116, Czech Republic; vaclav.dedic@mff.cuni.cz (V.D.); jan.franc@mff.cuni.cz (J.F.); eduard.belas@mff.cuni.cz (E.B.); rejhonm@karlov.mff.cuni.cz (M.R.); pavel.moravec@mff.cuni.cz (P.M.); 2 Crytur Ltd., Palackého 175, Turnov 51101, Czech Republic; tous@crytur.cz; 3 National Radiation Protection Institute, Bartoškova 28, Prague 14000, Czech Republic; josef.voltr@suro.cz

**Keywords:** CdZnTe, X-ray detector, high flux, polarization, de-polarization, Pockels effect

## Abstract

This paper describes an application of infrared light-induced de-polarization applied on a polarized CdZnTe detector working under high radiation fluxes. We newly demonstrate the influence of a high flux of X-rays and simultaneous 1200-nm LED illumination on the spectroscopic properties of a CdZnTe detector. CdZnTe detectors operating under high radiation fluxes usually suffer from the polarization effect, which occurs due to a screening of the internal electric field by a positive space charge caused by photogenerated holes trapped at a deep level. Polarization results in the degradation of detector charge collection efficiency. We studied the spectroscopic behavior of CdZnTe under various X-ray fluxes ranging between $5\times 10^{5}$ and $8\times 10^{6}$ photons per mm${}^{2}$ per second. It was observed that polarization occurs at an X-ray flux higher than $3\times 10^{6}$ mm${}^{-2}$·s${}^{-1}$. Using simultaneous illumination of the detector by a de-polarizing LED at 1200 nm, it was possible to recover X-ray spectra originally deformed by the polarization effect.

## 1. Introduction

High-resistivity CdZnTe (CZT) is a material of choice for high energy X-ray and gamma ray detectors working at room temperature due to the high average atomic number and wide bandgap (∼1.6 eV). Nowadays, CZT can find a use in several high radiation flux applications, such as computed tomography, gamma cameras, mammography and astrophysics [1]. The main factor usually limiting the charge collection efficiency (CCE) of CZT detectors under high radiation fluxes is the polarization phenomenon. It consists of a deformation of the internal electric field caused by an accumulation of positive space charge at deep levels due to the trapping of photogenerated holes [2,3]. In this case, the internal electric field in the detector strongly increases towards the cathode and decreases towards the anode. A region with a very low electric field is formed, which results in a reduction of CCE.

Our motivation of the infrared (IR) de-polarization of the detector is based on several studies of the high flux optical manipulation of deep level occupations in the bandgap, which were performed on CdTe [4,5] and CZT [6,7]. We have previously introduced the concept of the electric field restoration of polarized In-doped CZT detectors based on the optical transition of electrons from the valence band to the deep level in which the holes are trapped. This transition induced by the IR light with the wavelength around 1200 nm (∼1.03 eV) [8] reduces the originally positive space charge accumulation.

The aim of this paper is first to describe an application of IR light-induced de-polarization applied on a polarized CZT detector working under high radiation fluxes. Furthermore, we newly present the utilization of simultaneous IR illumination on the spectroscopic properties of a CZT detector operating under high flux radiation. The internal electric field study by Pockels effect measurements and the pulse height spectrum analysis using a standard spectroscopic setup were used for the characterization of the IR de-polarization phenomena. In some measurements, the LED at 940 nm (∼1.32 eV) instead of X-rays was used, because it was previously shown [9] that the IR light with a slightly below the bandgap wavelength (around 900–950 nm in the case of CZT) produces electrons and holes in the CZT detectors and may cause their radiation-induced polarization at high fluxes similar to X-rays. Similar flux dependencies of electric field profiles and detector currents were observed for both types of excitations [10].

In the context of this paper, ‘high flux’ means a sufficient intensity of the radiation under which the distribution of the internal electric field in the detector changes, while under ‘low flux’, it does not. High flux is represented by X-rays or high intensity light and low flux by a low activity ${}^{241}$Am gamma source (89 kBq) or low intensity light.

## 2. Experimental Section

For this study, we have used two neighboring detector-grade samples cut from the 〈111〉-oriented single-crystal wafer of In-doped Cd${}_{0.9}$Zn${}_{0.1}$Te with the electron mobility-lifetime product ${\left(\mu \tau \right)}_{e}=3\times 10^{-3}$ cm${}^{2}$·V${}^{-1}$ and a resistivity of $10^{10}\;\Omega$·cm.

Sample 1 with dimensions of 5×4.3×1.5 mm${}^{3}$ was used for the Pockels effect measurements to demonstrate the radiation-induced polarization and to find an optimum wavelength for the de-polarization of the given material. The surface of the sample was optically polished in order to be suitable for monitoring the transmittance distribution of the testing light during the Pockels effect measurements. The used surface treatment introduces high leakage current. Therefore, the sample was equipped with gold and indium planar electrodes on large opposite surfaces by evaporation, which act as the cathode and the anode, respectively. In this configuration, the indium anode helps to reduce the leakage current.

**Figure 1:**
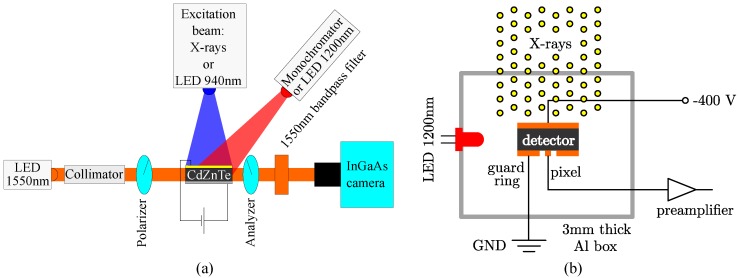
Experimental setup for Pockels effect measurements performed on Sample 1 (**a**). During standard electric field measurements, the cathode of the sample was irradiated by X-rays and simultaneously illuminated from the side by a 1200-nm LED during de-polarization mode. During the infrared spectral scanning (IRSS) measurements, the cathode was illuminated by a 940-nm LED and by tunable light from the monochromator from the side. The experimental setup used for X-rays measurements on Sample 2 (**b**). The planar cathode (top) was irradiated by X-rays. The pixel with a 0.5-mm diameter was surrounded by a guard ring covering the rest of the anode side (bottom).

Sample 2 with dimensions of 5×4×0.9 mm${}^{3}$ was used for X-ray and *γ*-ray spectroscopic measurements. Because there is no need for optically-polished surfaces to be used for spectroscopic measurements, its surface was chemically etched in a 1% Br-methanol solution for 1 min in order to reduce the leakage current. After the etching, the sample was equipped with evaporated gold contacts covering both larger sides. The gold contact, which was acting as the anode, was divided into a round pixel with a diameter of 0.5 mm and the surrounding guard ring separated by an approximately 0.5 mm-wide gap, as schematically shown in Figure 1b. The reason for using the pixel was to decrease the effective volume of the detector in order to reduce the number of events during the detection of the high flux of X-rays.

We have employed the Pockels electro-optic effect of CZT in order to measure the electric field distribution in Sample 1. It is a standard and widely-used method [11,12,13,14,15,16,17], which allows us to study samples under various excitation conditions. It is based on the collimated monochromatic low intensity light beam (1550 nm, ∼0.8 eV) passing through the biased sample (by high voltage supply ISEG SHQ 122M) placed between two orthogonal linear polarizers (Figure 1a). In this configuration, the spatial distribution of the transmittance $T\left(x,\;y\right)$ of the above-described system (monitored by an InGaAs camera) depends on the electric field distribution $E\left(x,\;y\right)$ in the sample as $T∼\sin^{2}E$.

The IR de-polarization using the LED of the specific wavelength of 1200 nm was motivated by the results of the influence of deep levels on the polarization- and de-polarization- related processes in detectors (see Section 3.1). Previously, for a study of the polarization and de-polarization processes in detectors, we developed the method of infrared spectral scanning (IRSS) exploiting the Pockels effect, which was tested on several CZT and CdTe samples [9,10,18]. The IRSS is based on measurements of the electric field profiles in the biased sample, which is excited by light with a fixed wavelength causing the polarization (represented by an LED at 940 nm in this paper). Then, it is simultaneously illuminated by a tunable IR light with wavelengths ranging between 0.9 and 1.7 μm (∼1.38–0.73 eV) coming from a Carl Zeiss SPM2 monochromator equipped with a 50-W halogen lamp and a LiF prism (Figure 1a). Tunable light from the monochromator with a constant photon flux is convenient for studying the change of the occupations of deep levels caused by the optical manipulation. By this method, it is possible to find an optimal de-polarizing wavelength.

During the X-ray measurements, samples were placed 1840 mm from a tungsten target X-ray tube MXR160 by GE Company with RQR6 standard radiation quality (80 kVp, 1 mm-thick inherent Be filtration and 3 mm-thick Al filter).

During spectroscopic measurements, the detector Sample 2 was placed inside a 3 mm-thick aluminum shielding box (Figure 1b). Its irradiated cathode was biased by high voltage supply ISEG SHQ 122M. Both the pixel and the guard ring were set to the zero potential, while the detector signal from the pixel was amplified with a preamplifier based on the Amptek A250 amplifier with a 560-μs decay time. The multichannel analyzer Canberra DSA1000 with the lowest rise time of 0.4 μs and zero flat top was used for spectral analysis. For ultra-high count rates, a lower rise time/flat top is useful, as the pile-up probability and the necessary processing ability decrease with decreasing rise time/flat top. The 1200-nm LED was used to illuminate the side of the detector from the distance of 1.5 cm. As the below bandgap light absorption of CZT is very low, we expect an almost homogeneous distribution of illumination by the light inside the whole detector sample. The forward current of the LED at 1200 nm was set to 50 mA, and its photon flux measured utilizing the Ophir Vega laser power meter with a Ge detector was $2\times 10^{14}\;{\mathrm{mm}}^{-2}$·s${}^{-1}$ during all of the measurements.

All of the measurements were performed at room temperature and in steady state conditions. The estimated error of the electric field measurements is 5%, and the error of the X-ray flux is 10%.

## 3. Results and Discussion

### 3.1. Polarization and IR De-Polarization

**Figure 2:**
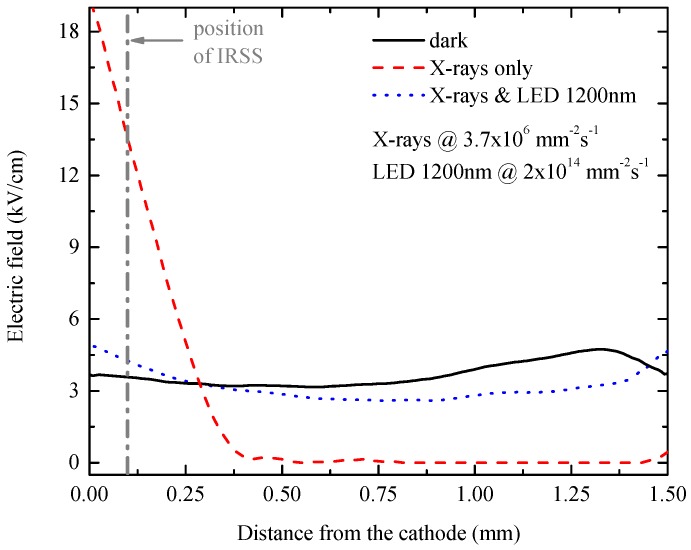
Electric field profiles between electrodes of planar detector Sample 1 biased at 500 V measured by the cross-polarizer technique based on the Pockels effect. The vertical dash-dotted line shows the depth below the cathode used for IRSS analysis.

The Pockels effect measurements were used in order to study the spectral dependence of radiation-induced polarization and IR de-polarization on the given CZT material. Sample 1 was biased at 500 V, while the gold was acting as the cathode, and its electric field profiles between electrodes were determined for various conditions (Figure 2). In the dark condition without any additional irradiation (solid line), the electric field is almost constant, which, according to the Gauss law, represents no or very low space charge accumulation. On the contrary, when the cathode side of the detector was irradiated by a high flux of X-rays of $3.7\times 10^{6}$ mm${}^{-2}$·s${}^{-1}$, the electric field was accumulated near the cathode and strongly decreased towards the anode forming the so-called dead layer with a very low electric field (dashed line). Such a situation is known as the radiation-induced polarization of the detector. It is caused by screening of the electric field by the positive space charge originating from photogenerated holes trapped at a deep level.

**Figure 3:**
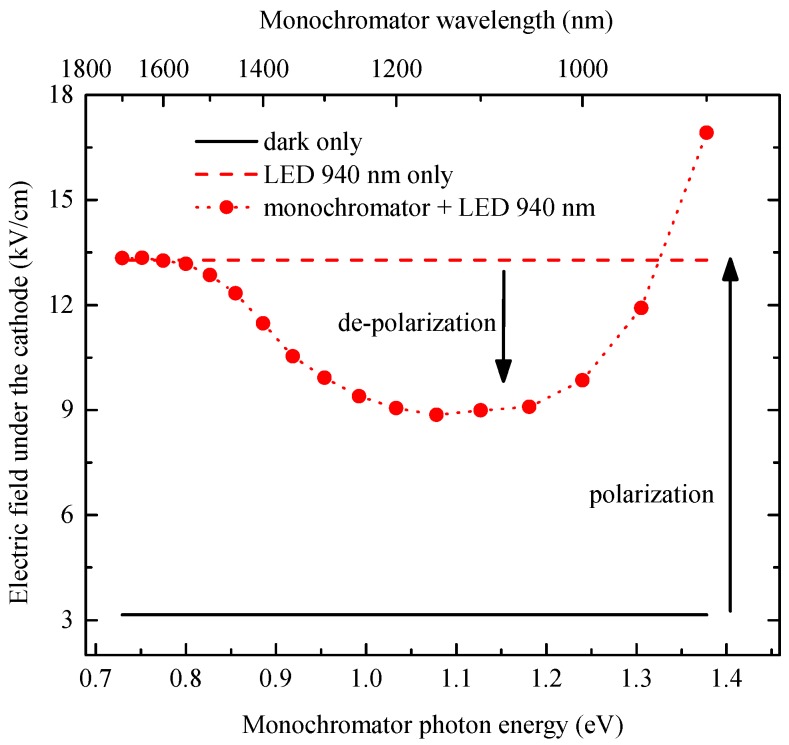
Infrared spectral scanning (IRSS) of the electric field under the cathode of planar detector Sample 1 biased at 500 V measured by the cross-polarizer technique based on the Pockels effect.

In order to find an optimal wavelength for the IR de-polarization, the IRSS measurements were performed at 500 V, and the evaluated electric field at the depth of 0.12 mm under the cathode is shown in Figure 3. In the dark condition, the electric field is ∼3.3 kV/cm, as can be expected from the applied bias (solid line in Figure 3). Then, the sample was set to the polarized state by the cathode side illumination with the 940-nm LED, and the electric field below the cathode increases to ∼13.3 kV/cm (dashed line in Figure 3). With simultaneous illumination of the detector side with low energy light from the monochromator (fixed flux of $5\times 10^{13}$ mm${}^{-2}$·s${}^{-1}$ for all of the wavelengths; circles in Figure 3) ranging between 0.73 eV (1700 nm) and 0.8 eV (1550 nm), there is no significant change of the electric field. Between 0.8 eV (1550 nm) and approximately 1.1 eV (1130 nm), there is a significant lowering (∼9 kV/cm) of the electric field below the cathode (decrease of the accumulated positive space charge). This IR light-induced de-polarization effect was explained in [8] as the neutralization of the positive space charge associated with trapped holes by an optical transition of electrons from the valence band. This de-polarization gets stronger with increasing intensity of the de-polarizing light. With a further increase of the photon energy of the light from the monochromator above 1.1 eV (wavelength below 1130 nm), the positive space charge accumulation prevails due to the trapping of a high amount of photogenerated holes originating in higher absorption when the photon energy approaches the main photoconductivity peak localized slightly below the bandgap energy [19]. The positive space charge accumulation could be also explained by the optical transition of electrons from the level with the energy of 1.1 eV to the conduction band [10]. Both mechanisms lead to the same effect of positive space charge accumulation in the case of the below bandgap light illumination.

Based on the strongest de-polarization effect around 1.05 eV (1180 nm) shown in Figure 3, we have chosen a commercially available LED with a central wavelength at 1200 nm (∼1.03 eV) for the demonstration of IR de-polarization. Moreover, the 1200-nm LED has higher optical power than 1200-nm light from a monochromator. IR de-polarization of the detector under a high flux of X-rays is demonstrated in Figure 2 (dotted line), where the electric field profile of Sample 1 under X-rays and simultaneous illumination with the 1200-nm LED is again almost flat, like in the dark condition, which is a sign of no significant space charge accumulation. The stronger effect of IR de-polarization by the 1200-nm LED than the monochromator (Figure 2) is caused by the four-times higher photon flux of the used LED.

### 3.2. IR De-Polarization in Spectroscopic Mode: X-rays

In this section of the paper, the results of X-rays’ spectroscopic measurements performed on the CZT Sample 2 biased at 400 V under X-rays are presented. The cathode of the sample was irradiated by high fluxes of X-rays, and simultaneously, the side of the sample was illuminated with an LED at 1200 nm. The experimental setup is shown in Figure 1b.

**Figure 4:**
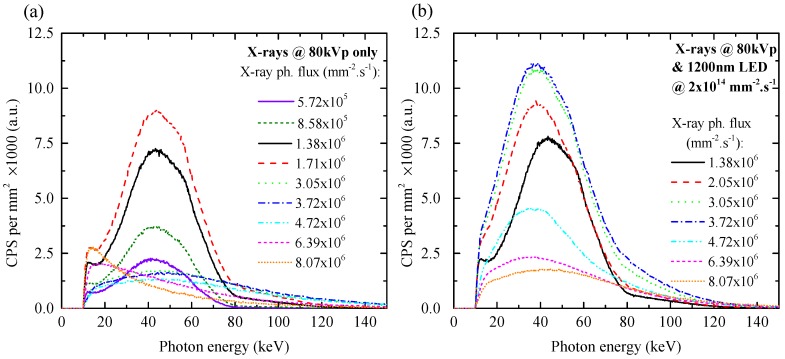
Spectra measured on the central pixel of Sample 2 under X-rays only (**a**) and under X-rays and simultaneous illumination with the 1200-nm LED with photon flux $2\times 10^{14}\;{\mathrm{mm}}^{-2}$·s${}^{-1}$ (**b**). The X-ray tube was biased at 80 kVp. The photon energy was calibrated using a ${}^{241}\mathrm{Am}$ gamma source.

Figure 4a shows the spectral response of Sample 2 under X-rays coming from an X-ray tube set to 80 kVp for various X-ray fluxes created by different X-ray tube powers. Under a low radiation flux, while the flux of the X-ray tube is set between $5.72\times 10^{5}$ mm${}^{-2}$·s${}^{-1}$ and $1.71\times 10^{6}$ mm${}^{-2}$·s${}^{-1}$, there is no polarization of the detector. The maximum of the spectral curve is located at around 40 keV and decreases to the lower energies because of the shielding by a 3 mm-thick Al box and a 3 mm-thick Al filter of RQR6. Although the photon energy should be limited to 80 keV, there is a tail at higher energies caused by pile-up events. A pile-up event occurs when the detection system’s readout electronics records two or more incident X-ray photons that are proximate in time, as only one photon with higher energy. Nevertheless, the amount of pile-up events is negligible, maximally up to 8% for energy higher than 80 keV during all of the measurements; therefore, we do not discuss this issue anymore, and the rest of the discussion is only about polarization and de-polarization effects.

**Figure 5:**
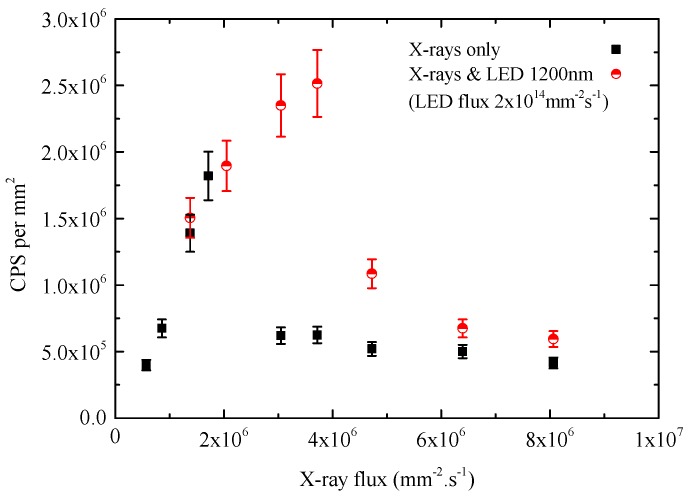
Sample 2: dependence of counts per second per mm${}^{2}$ on X-ray flux.

Figure 5 shows the total number of counts per second (CPS) per mm${}^{2}$ dependence on the X-ray flux recorded by the detecting system. For the X-ray-only measurements (squares in Figure 5), CPS increases linearly up to the flux of $1.71\times 10^{6}$ mm${}^{-2}$·s${}^{-1}$. For flux equal to and higher than $3.05\times 10^{6}$ mm${}^{-2}$·s${}^{-1}$, total CPS decreases due to worse CCE caused by polarization. Spectra from Figure 4a for X-ray flux equal to and higher than $3.05\times 10^{6}$ mm${}^{-2}$·s${}^{-1}$ lose their typical shape due to the polarization of the detector.

Figure 4b shows the spectra recorded by the detection system under X-rays and simultaneous illumination of the detector by the 1200-nm LED. It can be seen that the spectra are also slightly affected by the high energy tail of pile-up events, but they still keep their typical shape up to $3.72\times 10^{6}$ mm${}^{-2}$·s${}^{-1}$ of X-ray flux. Although there is a visible polarization at the flux of $4.72\times 10^{6}$ mm${}^{-2}$·s${}^{-1}$ demonstrated by the lower charge collection, the position of spectral maxima can more or less maintain their position compared to the case without the LED illumination. The influence of the 1200-nm LED on the energy resolution of the detector is discussed in Section 3.3. Comparing the measured X-ray spectra from Figure 4a,b, it is obvious that simultaneous de-polarizing illumination keeps the spectral information up to higher X-ray fluxes. This conclusion is also supported by Figure 5 (circles), where the number of counts increases for the X-ray flux up to $3.72\times 10^{6}$ mm${}^{-2}$·s${}^{-1}$. For higher X-ray fluxes, it decreases because of insufficiently high LED intensity; therefore, the polarization prevails again.

### 3.3. Energy Resolution: Gamma-rays

We tested the influence of IR de-polarization on the energy resolution of Sample 2 using *γ*-${}^{241}$Am. This gamma source with a low activity of 89 kBq was put inside the shielding Al box at a distance of 1 cm from the cathode of the detector. Polarization and de-polarization modes were activated using the LEDs at 940 and 1200 nm. The advantage of this method was the possibility to compare spectral resolution under various conditions with the measurements under X-rays in Section 3.2. In the case of X-rays, the detector spectral resolution was not sufficient to observe characteristic X-rays of tungsten K lines, and the bremsstrahlung had a broad spectrum, which was not sufficient for deciding about the detector energy resolution.

**Figure 6:**
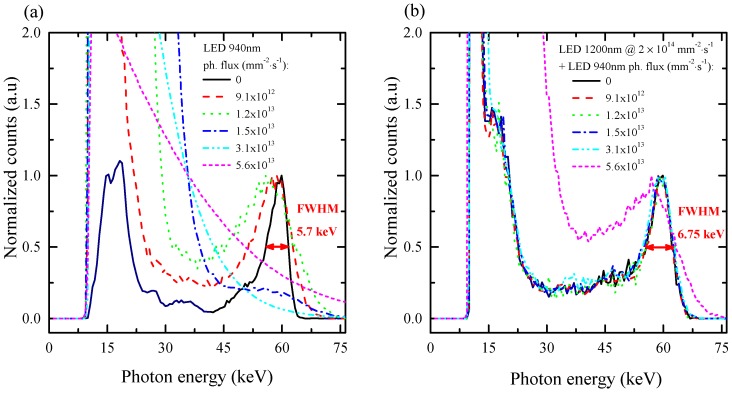
Pulse height spectra of *γ*-${}^{241}$Am obtained with Sample 2 under various levels of polarization by the LED at 940 nm (**a**) and with the additional depolarization (**b**), both at a 400-V bias. The spectra were normalized to the maximal number of counts for the photon energy equal to or higher than 35 keV. De-polarization was set by the LED at 1200 nm with photon flux of $2\times 10^{14}\;{\mathrm{mm}}^{-2}$·s${}^{-1}$.

Figure 6a shows the gamma spectra of *γ*-${}^{241}$Am under simultaneous illumination by the polarizing LED at 940 nm simulating a high flux of X-rays. The spectra were normalized to the maximal number of counts for the photon energy equal to or higher than 35 keV. This normalization was chosen in order to compare spectra around the 59.6-keV photopeak of ${}^{241}$Am. In the dark (gamma source only), the energy resolution of the used detector Sample 2 at 59.6 keV was 5.7 keV (∼9.6%), and the L line of ${}^{237}$Np (the product of the alpha decay of ${}^{241}$Am) is also apparent at around 17 keV. With increasing of the photon flux of the 940-nm LED, the energy resolution worsened significantly, and the counts at lower energies related to higher electronic noise of the spectroscopic apparatus increased. The higher the photon flux of the 940-nm LED is, the higher is the amount of photogenerated electrons, resulting in a larger current flowing through the whole spectroscopic apparatus. This current contributes to the noise of the used electronics (e.g., amplifier) [20], which is reflected in the low energy part of the spectrum. For the 940-nm LED flux higher than $1.5\times 10^{13}\;{\mathrm{mm}}^{-2}$·s${}^{-1}$, the photopeak completely disappeared due to the noise of the detector. Furthermore, in Figure 6a, it is visible that the higher detector polarization shifted the central position of the 59.6-keV photopeak to lower energies. This is caused by the losses of the photogenerated electrons due to the polarization.

On the other hand, gamma spectra from Figure 6b measured under simultaneous illumination of the sample by both polarizing (940 nm) and de-polarizing (1200 nm) LEDs do not change until a certain flux of $5.6\times 10^{13}\;{\mathrm{mm}}^{-2}$·s${}^{-1}$ of polarizing light. The photon flux of the de-polarizing LED at 1200 nm was fixed to $2\times 10^{14}\;{\mathrm{mm}}^{-2}$·s${}^{-1}$. Although the low channel noise is higher than in the dark condition (Figure 6a), the negative influence of the 1200-nm LED illumination on the spectra is lower at higher energies, and the detector energy resolution is 6.75 keV (∼11.3%) at 59.6 keV. This fact could make IR de-polarization useful for spectroscopic CZT detectors operating under high radiation fluxes.

## 4. Conclusions

We have demonstrated a promising mechanism of the IR de-polarization of spectroscopic In-doped CZT detectors operating under a high flux of X-rays using the LED at 1200 nm with a relatively high photon flux of $2\times 10^{14}\;{\mathrm{mm}}^{-2}$·s${}^{-1}$. This optimal de-polarizing wavelength was found using the IRSS method based on the internal electric field profile determination by the Pockels effect measurements. The IR de-polarization is based on the neutralization of a positive space charge in the detector by an optical transition of electrons from the valence band to the deep level. With the intensity of the used 1200-nm LED, the IR de-polarization works up to approximately a two-times higher X-ray flux than without the LED on the studied detector.

Although there is a quite high low-channel noise under the 1200-nm illumination, the energy resolution of the detector is only slightly affected at photon energies around 60 keV, which can make the concept of IR de-polarization useful. High flux spectroscopic radiation detectors with IR LED de-polarization could be successfully applied in nuclear power plants, computed tomography, X-ray defectoscopy, etc.

## Abbreviations

CCE	Charge collection efficiency
CZT	CdZnTe
IR	Infrared
IRSS	Infrared spectral scanning method
LED	Light-emitting diode
